# Utility of umbilical cord blood 25-hydroxyvitamin D levels for predicting bronchopulmonary dysplasia in preterm infants with very low and extremely low birth weight

**DOI:** 10.3389/fped.2022.956952

**Published:** 2022-08-04

**Authors:** Haoting Yu, Jianhua Fu, Yong Feng

**Affiliations:** Department of Pediatrics, Shengjing Hospital of China Medical University, Shenyang, China

**Keywords:** 25-hydroxyvitamin D, bronchopulmonary dysplasia, very low birth weight infants, extremely low birth weight infants, preterm infants

## Abstract

**Background and objective:**

There remains controversy regarding vitamin D deficiency and bronchopulmonary dysplasia (BPD) in very low birth weight (VLBW) and extremely low birth weight (ELBW) preterm infants. This study aimed to determine the prevalence of vitamin D deficiency assessed by umbilical cord blood 25-hydroxyvitamin D [25(OH)D] in preterm infants in northeast China and to evaluate the ability and optimal threshold of 25(OH)D for predicting BPD.

**Methods:**

The clinical data of VLBW and ELBW preterm infants with known cord-blood 25(OH)D levels were analyzed retrospectively. Infants were divided into groups based on their cord-blood 25(OH)D levels and BPD diagnosis. Logistic regression was performed to assess the risk factors for BPD and a nomogram was established. Receiver operating characteristic (ROC) curve analysis was used to evaluate the optimal threshold of cord-blood 25(OH)D concentration for predicting BPD.

**Results:**

A total of 267 preterm infants were included, of which 225 (84.3%) exhibited vitamin D deficiency and 134 (50.2%) were diagnosed with BPD. The incidence of BPD was lower in the group with a 25(OH)D level of >20 ng/ml than in the other groups (*P* = 0.024). Infants with BPD had lower cord-blood 25(OH)D levels than those without BPD (11.6 vs. 13.6 ng/ml, *P* = 0.016). The multivariate logistic regression model revealed that 25(OH)D levels (odds ratio [OR] = 0.933, 95% confidence interval [95% CI]: 0.891–0.977), gestational age (OR = 0.561, 95% CI: 0.425–0.740), respiratory distress syndrome (OR = 2.989, 95% CI: 1.455–6.142), and pneumonia (OR = 2.546, 95% CI: 1.398–4.639) were independent risk factors for BPD. A predictive nomogram containing these four risk factors was established, which had a C-index of 0.814. ROC curve analysis revealed that the optimal cutoff value of 25(OH)D for predicting BPD was 15.7 ng/ml (area under the curve = 0.585, 95% CI: 0.523–0.645, *P* = 0.016), with a sensitivity of 75.4% and a specificity of 42.9%.

**Conclusions:**

A cord-blood 25(OH)D level of <15.7 ng/ml was predictively valuable for the development of BPD. The nomogram established in this study can help pediatricians predict the risk of BPD more effectively and easily.

## Introduction

Bronchopulmonary dysplasia (BPD) is one of the commonest as well as one of the most serious respiratory complications in preterm infants. Recent years have seen the gradual evolution of perinatal medicine and neonatal intensive care, resulting in improved survival rates among very low birth weight (VLBW) and extremely low birth weight (ELBW) preterm infants (1). However, BPD remains a serious challenge in preterm care, affecting approximately one-quarter of VLBW and half of ELBW preterm infants (2). Early detection and intervention can help reduce the incidence of BPD and improve the prognosis and quality of life of patients. However, although several biomarkers have been developed to predict the development of BPD, none of them have been validated (3, 4). As such, these are not appropriate for use in clinical practice.

Vitamin D is a sterol hormone, whose active form (and best marker) is 25-hydroxyvitamin D [25(OH)D] (5). Vitamin D deficiency is common both in pregnant women (6, 7) and in preterm infants (8–13). Approximately 37–92% of preterm infants have been reported to suffer from vitamin D deficiency due to their short gestational age and reduced maternal vitamin D supplementation during pregnancy (8–13). In addition, vitamin D deficiency at birth is associated with poor respiratory outcomes—especially BPD—in preterm infants (13–16). Vitamin D participates in cell proliferation, cell differentiation, and the regulation of fetal lung maturation, whereas vitamin D deficiency has been hypothesized to aggravate lung diseases in premature neonates (17, 18). The association between vitamin D deficiency and the development of BPD is controversial (8–10, 12, 14–16). As such, the widespread incidence of vitamin D deficiency may be due to between-study differences in cutoffs for vitamin D deficiency, gestational age, and maternal vitamin D status during pregnancy. However, few studies have investigated the incidence of vitamin D deficiency and its association with the development of BPD in VLBW and ELBW preterm infants. Such studies are especially lacking in northeast China, where insufficient sunlight coincides with a high incidence of vitamin D deficiency in pregnant women (19).

Vitamin D deficiency is a condition that can be prevented during pregnancy and early postnatal life. Moreover, vitamin D deficiency at birth has been suggested as a potential biomarker of BPD. A recent study demonstrated that early vitamin D supplementation can prevent BPD and improve clinical outcomes (20); however, the threshold for vitamin D supplementation in premature infants remains unclear. Therefore, the present study aimed to determine the incidence of vitamin D deficiency—as assessed using umbilical cord blood 25(OH)D levels—in VLBW and ELBW preterm infants in Shenyang, a typical city in northeast China (latitude, 41°48′N; longitude, 123°25′E). We also evaluated the optimal threshold of cord-blood 25(OH)D levels for predicting the development of BPD in VLBW and ELBW preterm infants.

## Methods

### Subjects

In this retrospective study, we analyzed data collected from preterm infants in the neonatal intensive care unit (NICU) of Shengjing Hospital, China, between June 2016 to June 2021. The inclusion criteria for infants were as follows: (1) gestational age <32 weeks, (2) VLBW (birth weight <1,500 g) and ELBW (birth weight <1,000 g) preterm infants, and (3) infants whose cord-blood 25(OH)D levels had been measured. The exclusion criteria for infants were as follows: (1) severe congenital malformations, genetic and metabolic diseases, chromosomal diseases, or other organ function-related emergencies; (2) mothers with severe liver, kidney, or thyroid diseases; (3) abandonment of treatment during hospitalization; and (4) lack of complete records for gestational age, birth weight, or clinical data. This study was conducted in accordance with the World Medical Association's Declaration of Helsinki and was approved by the local Ethics Committee (ethical examination approval number: 2021PS891K).

### Measurement of 25(OH)D levels in umbilical cord blood

Umbilical cord blood collected from neonates immediately after birth and the samples were immediately delivered to the Clinical Test Center of Shengjing Hospital. The 25(OH)D concentrations (expressed in ng/ml) were measured with electrochemiluminescence using the Cobas e601 module (Roche Diagnostics International AG, Rotkreuz, Switzerland) according to the manufacturer's instructions. Based on the cord-blood 25(OH)D levels (21–25), the infants were divided into four groups: >20 ng/ml (*n* = 42), >15–20 ng/ml (*n* = 54), >10–15 ng/ml (*n* = 79), and ≤ 10 ng/ml (*n* = 92). Vitamin D deficiency was defined as a 25(OH)D level of ≤ 20 ng/ml.

### Disease definitions

BPD was diagnosed in preterm infants based on the radiographic confirmation of parenchymal lung disease and the requirement of inhaled oxygen for >3 consecutive days to maintain an arterial oxygen saturation of 90–95% at 36 weeks' postmenstrual age (26). Based on the BPD diagnosis, the infants were divided into two groups: BPD (*n* = 134) and non-BPD (*n* = 133). Respiratory distress syndrome (RDS) was diagnosed based on the signs of dyspnea (retractions, grunting, or flaring), increased oxygen requirement, and typical chest radiographic features (fine granular densities, bronchial bronchograms, ground-glass opacity, or white lungs) (27). Neonatal pneumonia was diagnosed based on a combination of radiographic evidence, worsening gas exchange, and clinical and/or laboratory evidence. Radiographic evidence included new or progressive infiltrate, consolidation, cavitation or pneumoatocele. Clinical and/or laboratory findings included cough, wheezing, signs of dyspnea, rales, increased respiratory secretions, temperature instability, leukopenia or leukocytosis (28, 29).

### Ventilation protocol

During the study period, we used the protocol on ventilation suggested by the Chinese guideline published in May 2015 (30). The intubation criteria were as follows: (1) frequent apneas, unresponsive to medication or non-invasive ventilation; (2) RDS required surfactant administration; (3) fraction of inspired oxygen (FiO_2_) > 0.6–0.7, arterial partial pressure of oxygen <50–60 mmHg, or transdermal oxygen saturation <85%; (4) arterial partial pressure of carbon dioxide > 60–65 mmHg, with persistent acidosis (pH <7.20). When the intubated infants got clinical stability and normal blood gas values, the ventilator settings were gradually adjusted. If an infant met all of the following criteria, extubation was attempted: (1) peak inspiratory pressure ≤ 18 cm H_2_O; (2) positive end-expiratory pressure at 2–4 cm H_2_O; (3) breathing rate ≤ 10 breaths/min; (4) FiO_2_ ≤ 0.4; (5) normal blood gas values. For ventilator-dependent infants with postnatal age > 12–14 days and FiO_2_ > 0.6, low-dose dexamethasone was used for <7 days to facilitate extubation. Infants with RDS were treated with surfactant according to the European Consensus Guidelines on the Management of Neonatal Respiratory Distress Syndrome in Preterm Infants—2013 Update (31).

### Nutritional protocol

During the study period, the nutritional protocol for preterm infants was based on the Chinese guideline published in October 2013 (32). Parenteral nutrition (PN) was started within 24 h after birth and individualized PN was prescribed daily. Vitamin D was added into PN at 32 IU/kg/day. PN was given to infants until enteral nutrition (EN) calorie intake reached 80 kcal/kg/day. EN was initiated within 12 h after birth for infants with a birth weight of more than 1,000 g and delayed until 24–48 h after birth for those with severe perinatal asphyxia, umbilical arterial cannula and those with a birth weight of <1,000 g. Infants without contraindications to EN support received minimal enteral nutrition as early as possible after birth, with breast milk or formula. Human milk fortifier was added for infants with birth weight <2,000 g when feeding volume reached 50–100 ml/kg/day. Human milk fortifier contained about 1.5 IU/ml vitamin D and formula contained about 1.25 IU/ml vitamin D. Fortified breast milk contains roughly the same amount of vitamin D as formula. Moreover, all preterm infants without contraindications to EN support were treated with oral vitamin D (800 IU/day) starting at 15 days of life.

### Data collection

Electronic medical records were examined and the demographic and clinical characteristics of the subjects were collated. The data included information on sex, gestational age, mode of delivery, birth weight, respiratory outcomes, Apgar score at 1 min, initiation and duration of invasive mechanical ventilation, duration of mechanical ventilation (invasive and non-invasive), duration of low-flow oxygen, discharged on home oxygen, length of NICU, and length of hospital stay of VLBW and ELBW preterm infants.

### Statistical analysis

The data distributions were assessed using the Shapiro–Wilk test. Normally distributed variables were expressed as the mean ± standard deviation. Analysis of variance (ANOVA) and Student's *t*-test were used to evaluate the differences among four groups with different 25(OH)D levels and between the BPD and non-BPD groups, respectively. Non-normally distributed variables were expressed as the median (interquartile range [IQR]), and between-group differences were analyzed using the Mann–Kruskal–Wallis *H*-test and Whitney *U*-test, as appropriate. Categorical variables were expressed as percentages and compared using the chi-square test. Logistic regression analysis was performed to evaluate the risk factors for BPD in VLBW and ELBW preterm infants, and the factors included in the final analysis were determined based on their statistical significance and clinical values. A nomogram was established based on the multivariate regression model. The regression coefficients were converted into scores on a 100-point scale (range, 0–100), and the scores for each risk factor were added to calculate the total score. The concordance index (C-index) was measured to assessed the discrimination performance of the nomogram. The calibration curve and Hosmer–Lemeshow test were conducted to assess the calibration of the nomogram. Receiver operating characteristic (ROC) curves and areas under the curve (AUC) were used to assess the predictive and cutoff values of 25(OH)D level for BPD. Two-tailed *P*-values <0.05 were considered statistically significant. Statistical analysis was performed using R version 4.2.0 (R Foundation for Statistical Computing, Vienna, Austria) and MedCalc version 20.014 (MedCalc Software Ltd., Acacialaan, Ostend).

## Results

### Demographic and clinical characteristics stratified by cord-blood 25(OH)D level

Table 1 lists the demographic and clinical characteristics of VLBW and ELBW preterm infants stratified by cord-blood 25(OH)D levels. We retrospectively evaluated the data of 267 preterm infants (141 boys and 126 girls). In total, 225 (84.3%) infants had vitamin D deficiency. Of these, 54, 70, and 92 had 25(OH)D levels >15–20, >10–15, and ≤ 10 ng/ml, respectively. There were no significant differences in the demographic characteristics of infants, including sex, gestational age, mode of delivery, and birth weight. Moreover, there were no significant differences among groups with different cord-blood 25(OH)D levels in terms of Apgar score at 1 min, proportions of RDS and pneumonia, respiratory supports, length of NICU stay, and length of hospital stay. However, incidences of BPD were lower in the group with >20 ng/ml 25(OH)D than in the groups with other 25(OH)D levels (*P* = 0.024).

**Table 1:** Demographic and clinical characteristics of preterm infants grouped by cord-blood 25(OH)D levels (ng/ml).

**Characteristics**	>**20 (*****n*** = **42)**	>**15–20 (*****n*** = **54)**	>**10–15 (*****n*** = **79)**	≤ **10 (*****n*** = **92)**	***P*** **-value**
Sex (% male)	16 (38.1%)	33 (61.1%)	43 (54.4%)	49 (53.3%)	0.157
Gestational age (weeks)^a^	29.4 ± 1.3	29.1 ± 1.2	29.4 ± 1.3	29.4 ± 1.2	0.583
Cesarean section	30 (71.4)	39 (72.2)	58 (73.4)	61 (66.3)	0.758
Birth weight (g)^a^	1155.0 ± 225.9	1164.0 ± 207.7	1186.5 ±178.6	1177.9 ± 187.5	0.826
ELBW	10 (23.8%)	12 (22.2%)	14 (17.7%)	16 (17.4%)	0.760
VLBW	32 (76.2%)	42 (77.8%)	65 (82.3%)	76 (82.6)	
Apgar score at 1 min	8.0 (5.8–9.0)	7.0 (6.0–9.0)	8.0 (6.0–9.0)	8.0 (7.0–9.0)	0.948
Respiratory outcomes
RDS	23 (54.8%)	41 (75.9%)	55 (74.3%)	64 (69.6%)	0.162
Pneumonia	19 (45.2%)	32 (59.3%)	36 (48.6%)	43 (46.7%)	0.387
BPD	13 (31.0%)	25 (46.3%)	42 (53.2%)	54 (58.7%)	0.024
Respiratory supports
Initiation of invasive MV
No	9 (21.4%)	12 (22.2%)	9 (11.4%)	18 (19.6%)	0.323
≤ 24 h after birth	25 (59.5%)	28 (51.9%)	53 (67.1%)	59 (64.1%)	0.320
>24 h after birth	8 (19.0%)	14 (25.9%)	17 (21.5%)	15 (16.3%)	0.555
Duration of MV (days)	23.8 (10.8–36.1)	30.9 (17.1–43.1)	25.8 (14.1–39.3)	25.2 (16.6–35.4)	0.344
Invasive MV (days)	4.4 (1.3–9.1)	7.0 (1.2–13.1)	5.7 (2.0–18.3)	7.6 (1.42–15.0)	0.276
Duration of low-flow oxygen (days)	8.9 (5.8–16.0)	9.7 (3.6–15.1)	10.4 (4.8–17.0)	13.1 (6.1–18.7)	0.263
Discharged on home oxygen	6 (19.0%)	13 (24.1%)	9 (11.4)	14 (15.2)	0.265
Length of NICU stay (days)	24.5 (17.0–38.3)	30.0 (16.8–42.3)	26.0 (18.0–37.0)	26.0 (17.0–35.0)	0.576
Length of hospital stay (days)	50.5 (43.5–60.8)	55.0 (43.0–66.0)	55.0 (43.0–69.0)	54.0 (42.0–62.5)	0.749

*Except where noted, continuous variables have been expressed as median (IQR), and corresponding P-values have been obtained from the Kruskal–Wallis H-test. Categorical variables have been expressed as n (%), and corresponding P-values have been determined using the chi-square test.*

*RDS, respiratory distress syndrome; BPD, bronchopulmonary dysplasia; MV, mechanical ventilation; NICU, neonatal intensive care unit.*

*^a^Expressed in mean ± standard deviation, and the P-values have been obtained from an analysis of variance*.

### Demographic and clinical characteristics of BPD and non-BPD infants

Table 2 lists the results of comparative analyses of demographic and clinical characteristics between BPD and non-BPD infants. The sex and mode of delivery were comparable between the BPD and non-BPD groups. Compared to non-BPD infants, BPD infants had significantly lower gestational age, birth weight, and Apgar score at 1 min (all *P* < 0.05). The median cord-blood 25(OH)D level was lower in the BPD group than that in the non-BPD group (11.6 vs. 13.6 ng/ml, *P* = 0.016). Furthermore, compared with the non-BPD infants, BPD infants exhibited significantly higher frequencies of RDS, pneumonia, invasive mechanical ventilation initiated within 24 h after birth, and discharge on home oxygen, as well as longer durations of mechanical ventilation, low-flow oxygen, NICU stay, and hospital stay (all *P* < 0.05).

**Table 2:** Demographic and clinical characteristics of infants with and without bronchopulmonary dysplasia (BPD).

**Characteristics**	**BPD group (*****n*** = **134)**	**Non-BPD group (*****n*** = **133)**	***P*** **-value**
Sex (% male)	77 (57.5%)	64(48.1%)	0.142
Cesarean section	91 (67.9%)	97 (72.9%)	0.422
Gestational age (weeks)^a^	28.9 ± 1.1	29.8 ± 1.2	<0.001
Birth weight (g)^a^	1118.9 ± 205.6	1229.6 ± 166.6	<0.001
ELBW	37 (27.6%)	15 (11.3%)	0.001
VLBW	97 (72.4%)	118 (88.7%)	
Cord-blood 25(OH)D (ng/ml)	11.6 (8.5–15.7)	13.6 (8.9–19.4)	0.016
Apgar score at 1 min	7.0 (6.0–9.0)	8.0 (7.0–10.0)	0.003
Respiratory outcomes
RDS	113 (84.3%)	70 (52.6%)	<0.001
Pneumonia	87 (64.9%)	43 (32.3%)	<0.001
Respiratory supports
Initiation of invasive MV
No	9 (6.7%)	39 (39.3%)	<0.001
≤ 24 h after birth	103 (76.9%)	62 (46.6%)	<0.001
>24 h after birth	22 (16.4%)	32 (24.1%)	0.120
Duration of MV (days)	34.3 (24.5–50.1)	17.3 (8.5–28.3)	<0.001
Invasive MV (days)	12.8 (7.2–22.7)	2.5 (0.0–6.0)	<0.001
Duration of low-flow oxygen (days)	15.1 (10.7–20.5)	6.3 (1.6–10.9)	<0.001
Discharged on home oxygen	40 (29.9%)	2 (1.5%)	<0.001
Length of NICU stay (days)	32.5 (25.0–46.0)	21.0 (12.5–28.0)	<0.001
Length of hospital stay (days)	59.0 (51.0–72.0)	46.0 (39.0–56.0)	<0.001

*Except where noted, continuous variables have been expressed as median (IQR), and corresponding P-values have been obtained from the Mann–Whitney U-test. Categorical variables have been expressed as n (%), and corresponding P-values have been determined using the chi-square test.*

*RDS, respiratory distress syndrome; MV, mechanical ventilation; NICU, neonatal intensive care unit.*

*^a^Expressed in mean ± standard deviation, and the P-values have been obtained from an Student's t-test*.

### Factors associated with BPD in VLBW and ELBW infants

**Figure 1:**
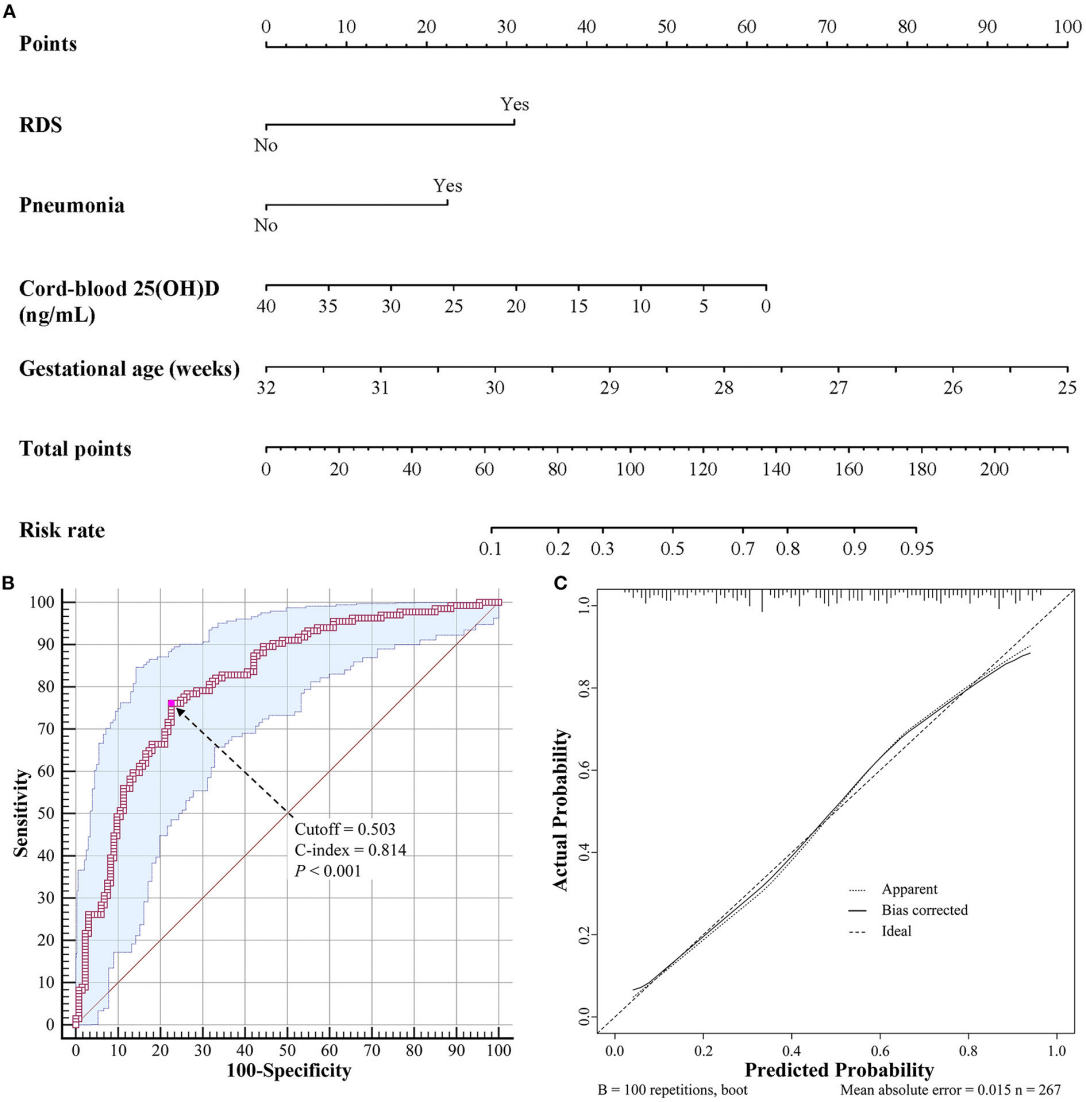
Nomogram for predicting BPD in VLBW and ELBW preterm infants. **(A)** Nomogram model for prediction of BPD. **(B)** Receiver operating characteristic curve of the nomogram model. **(C)** Calibration curve of the nomogram model. BPD, bronchopulmonary dysplasia; VLBW, very low birth weight; ELBW, extremely low birth weight.

Univariate and multivariate logistic regression models were used to explore the factors associated with BPD (Table 3). In the multivariate model, cord-blood 25(OH)D level (odds ratio [OR] = 0.933, 95% confidence interval [95% CI]: 0.891–0.977, *P* = 0.003), gestational age (OR = 0.561, 95% CI: 0.425–0.740, *P* < 0.001), RDS (OR = 2.989, 95% CI: 1.455–6.142, *P* = 0.003), and pneumonia (OR = 2.546, 95% CI: 1.398–4.639, *P* = 0.002) were independent risk factors associated with BPD. Birth weight, Apgar score at 1 min, and invasive mechanical ventilation did not contribute significantly to the model. Based on these results, we established a predictive nomogram containing these four risk factors (Figure 1A). The C-index was 0.814 (95% CI: 0.763–0.866), and the cutoff value was 0.503 (Figure 1B), with a sensitivity of 76.1%, specificity of 77.4%, positive likelihood ratio (LR) of 3.4, and negative LR of 0.3. These findings indicated the good predictive accuracy of the nomogram model, and the Hosmer–Lemeshow test resulted in a *P*-value of 0.188. Accordingly, the calibration curve of the nomogram showed good agreement for this cohort (Figure 1C).

**Table 3:** Factors associated with bronchopulmonary dysplasia (BPD) in very low birth weight (VLBW) and extremely low birth weight (ELBW) preterm infants.

**Factors**		**Univariate analysis**		**Multivariate analysis**	
		**OR (95%CI)**	***P*** **-value**	**OR (95%CI)**	***P*** **-value**
Sex	Male	1 (ref)		–	
	Female	1.456 (0.899–2.360)	0.127	–	
Cesarean section	No	1 (ref)		–	
	Yes	0.785 (0.464–1.331)	0.369	–	
Gestational age	Per week	0.474 (0.371–0.608)	<0.001	0.561 (0.425–0.740)	<0.001
Birth weight	ELBW	3.001 (1.555–5.790)	0.001	1.340 (0.604–2.970)	0.471
	VLBW	1 (ref)			
Apgar score at 1 min		0.845 (0.748–0.955)	0.007	0.952 (0.817–1.109)	0.530
Cord-blood 25(OH)D	Per ng/ml	0.954 (0.919–0.991)	0.026	0.933 (0.891–0.977)	0.003
RDS	No	1 (ref)		1 (ref)	
	Yes	4.843 (2.720–8.622)	<0.001	2.989 (1.455–6.142)	0.003
Pneumonia	No	1 (ref)		1 (ref)	
	Yes	3.874 (2.332–6.438)	<0.001	2.546 (1.398–4.639)	0.002
Initiation of invasive MV
No	0	1 (ref)		1 (ref)	
≤ 24 h after birth	1	7.679 (3.681–16.019)	<0.001	2.184 (0.858–5.563)	0.101
>24 h after birth	2	2.875 (1.225–6.746)	0.015	1.851 (0.674–5.085)	0.232

*OR, odds ratio; CI, confidence interval; RDS, respiratory distress syndrome; MV, mechanical ventilation*.

### Utility of cord-blood 25(OH)D levels for the prediction of BPD

**Figure 2:**
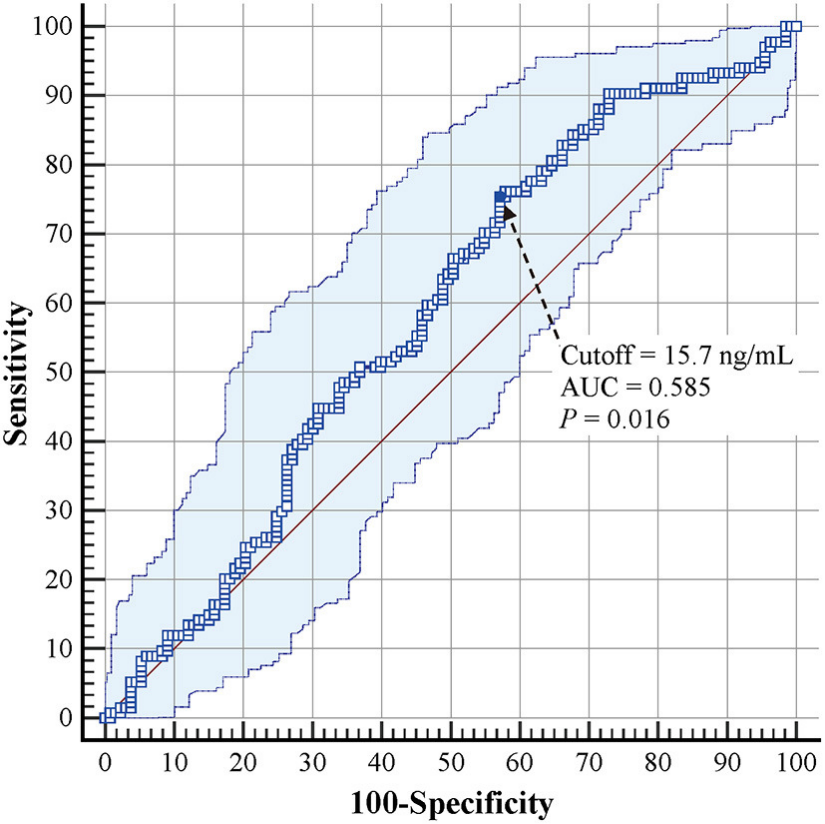
Receiver operating characteristic curves for cord-blood 25(OH)D levels in predicting BPD development in VLBW and ELBW preterm infants. BPD, bronchopulmonary dysplasia; VLBW, very low birth weight; ELBW, extremely low birth weight.

The predictive value of cord-blood 25(OH)D level was further assessed using ROC curves. The cutoff value of 25(OH)D level for predicting BPD development in VLBW and ELBW preterm infants was 15.7 ng/ml (AUC = 0.585, 95% CI: 0.523–0.645, *P* = 0.016) (Figure 2). The sensitivity, specificity, positive LR, and negative LR were 75.4%, 42.9%, 1.3, and 0.6, respectively. The positive predictive value was 57.1% (95% CI: 52.7–61.3%), and the negative predictive value was 63.3% (95% CI: 54.8–71.1%) in VLBW and ELBW infants.

## Discussion

In this retrospective study on cord-blood 25(OH)D levels in infants, we detected vitamin D deficiency ( ≤ 20 ng/ml) in 84.3% of VLBW and ELBW preterm infants in Shenyang. Based on our findings, we established an effective nomogram incorporating cord-blood 25(OH)D level, gestational age, RDS, and pneumonia to predict BPD in VLBW and ELBW preterm infants. Furthermore, a cord-blood 25(OH)D level of <15.7 ng/ml indicated the development of BPD and the need for early vitamin D supplementation. These findings may help prevent BPD and improve the clinical outcomes of preterm infants (20).

Vitamin D deficiency is very common in VLBW and ELBW preterm infants, especially in northeast China. The incidence of vitamin D deficiency in VLBW and ELBW infants was 84.3% in the present study, and has been reported to range from 37 to 92% in previous studies (8–13). Fetal and newborn cord-blood 25(OH)D concentrations depend on and are correlated with maternal 25(OH)D levels. This is because the fetus has no endogenous production mechanism for 25(OH)D and relies solely on transplacental transfer, mainly in the third trimester (33). The high incidence of vitamin D deficiency in our study may be due to the short gestational ages of the included infants. However, consistent with the findings of Kim et al. (13), we also failed to find any correlation between cord-blood 25(OH)D levels and gestational age. This may be because there were no full-term infants or infants with gestational ages > 32 weeks included in this study. Maternal vitamin D deficiency may also account for the high incidence of vitamin D deficiency. Kassai et al. (34) demonstrated that the 25(OH)D levels of newborns directly correlate with those of pregnant women. Exposure of the skin to ultraviolet-B is known to be one of the main sources of vitamin D. This study was conducted in a city in northeast China that experiences a cold and windy winter—conditions that reduce outdoor activities and sunshine exposure (19). Therefore, it is possible that vitamin D deficiency was common in the pregnant mothers in our study. A study conducted in Bucheon (a city in northern Korea) also reported that 79.8% of preterm infants had vitamin D deficiency (<20 ng/ml) (13), which is similar to the findings of our study. So, it is important to monitor vitamin D level for pregnant women and they should have more sun exposure and additional vitamin D intake to maintain normal vitamin D levels, which may be beneficial for the prevention of postnatal vitamin D deficiency.

Vitamin D deficiency is an independent risk factor for BPD in VLBW and ELBW preterm infants. Consistent with previous studies (13–16), we found that lower cord-blood 25(OH)D levels were an independent risk factor for BPD in VLBW and ELBW preterm infants. The main pathophysiology of BPD is lung injury (characterized by disrupted alveolarization and microvascular development) and also involves inflammation and immune response (26, 35). Animal studies have reported the potential biological roles of vitamin D in alveolar and vascular development—including in the differentiation of type II pneumocytes, lung mesenchymal stem cells, and vascular endothelial cells—and in the synthesis and secretion of surfactant phospholipids (36–38). In addition, vitamin D also modulates immune processes and the proliferation and differentiation of numerous other cell types (39). As a result, vitamin D deficiency contributes to several respiratory complications and lung function impairments in premature infants, including airway hyperresponsiveness, increased resistance, and reduced compliance (15, 40). However, the association between vitamin D status and the development of BPD remains controversial in clinical research due to the multifactorial etiology of BPD (8, 9, 12, 41). Moreover, vitamin D deficiency is not the sole risk factor for BPD, and the risk factors and infants included in analysis vary across studies. Consistent with previous findings (10, 42, 43), we demonstrated that lower gestational age, RDS, and pneumonia were additional risk factors for BPD. Based on these results, we established a nomogram containing cord-blood 25(OH)D level as a factor to predict BPD in VLBW and ELBW preterm infants.

There is still a lack of a suitable threshold to define vitamin D deficiency in VLBW and ELBW preterm infants. Most medical societies and organizations recommend a threshold of 20 ng/ml (22, 23, 44–46), whereas the Global Consensus for rickets (24) and the British Paediatric and Adolescent Bone Group (25) suggest cutoff values of 12 and 10 ng/ml, respectively. These thresholds were defined with respect to a general population with adequate bone health. For preterm infants, the American Academy of Pediatrics (47) and ESPGHAN (48) guidelines also recommended a threshold of 20 ng/ml, it was still with respect to adequate bone health. The sources of vitamin D for preterm infants mainly included parenteral nutrition, preterm infant formulas, and oral supplementation. ESPGHAN guidelines recommended a wide dose range of vitamin D (200–1,000 IU/day) for preterm infants on parenteral nutrition, which may be due to lack of optimum vitamin D requirements. The regular oral supplementation dose of 400 IU/day may be inadequate for preterm infants with high risk of BPD. Therefore, an adequate 25(OH)D threshold for vitamin D treatment in preterm infants for preventing BPD is warranted. A recent study showed that 800 IU/day of vitamin D administered within 48 h after birth reduced the incidence of BPD in preterm infants (20). However, Fort et al. (49) failed to demonstrate the preventive effects of vitamin D supplementation on BPD development, likely due to the different vitamin D statuses of the included infants in the two studies (20, 49). The present study demonstrates that a cord-blood 25(OH)D level of <15.7 ng/ml is an appropriate threshold for predicting BPD in VLBW and ELBW preterm infants. Moreover, the nomogram containing cord-blood 25(OH)D level can help pediatricians identify infants at high risk of BPD, which can help them make clinical decisions about the timing and dose of vitamin D supplementation.

This study had several limitations due to its descriptive nature and retrospective design. First, due to the retrospective study design, data on postnatal vitamin D status in the first 28 days after birth were unavailable and could not be compared between the BPD and non-BPD groups. However, all the included infants received similar nutritional protocol and oral vitamin D supplementation. The association between postnatal vitamin D status and BPD development is controversial and needs to be further investigated (20, 49, 50). Second, we excluded VLBW and ELBW infants whose cord-blood levels of 25(OH)D had not been measured, which may have introduced a selection bias. Nevertheless, the incidence of BPD in VLBW and ELBW infants was 50.2% in our study, which is similar to that reported in the literature (2). Third, there was a lack of data on maternal vitamin D supplementation and levels during pregnancy, which could help explore the causes of vitamin D deficiency in preterm infants and provide strategies for prevention. Finally, the threshold of cord-blood 25(OH)D level, in combination with the nomogram established in our study, should be validated in further prospective studies.

In conclusion, vitamin D deficiency was found to be very common in VLBW and ELBW preterm infants in northeast China. A cord-blood 25(OH)D level of <15.7 ng/ml was predictively valuable for BPD in VLBW and ELBW preterm infants, and the nomogram established in this study can help pediatricians predict the risk of BPD more effectively and easily during early postnatal life. Further prospective studies are warranted to validate the utility of the 25(OH)D threshold and the nomogram for early vitamin D supplementation and the prevention of BPD.

## Data availability statement

The raw data supporting the conclusions of this article will be made available by the authors, without undue reservation.

## Ethics statement

The studies involving human participants were reviewed and approved by Shengjing Hospital of China Medical University. Written informed consent from the participants' legal guardian/next of kin was not required to participate in this study in accordance with the national legislation and the institutional requirements.

## Author contributions

HY and YF conceived and designed the study, and interpreted the data and drafted the manuscript which was revised by all authors. HY and JF were responsible for the collection and analysis of the experimental data. All authors read and approved the final manuscript.

## Funding

This study was funded by the Key R&D Guidance Plan Projects of Liaoning Province (2020JH1/10300001).

## Conflict of interest

The authors declare that the research was conducted in the absence of any commercial or financial relationships that could be construed as a potential conflict of interest.

## Publisher's note

All claims expressed in this article are solely those of the authors and do not necessarily represent those of their affiliated organizations, or those of the publisher, the editors and the reviewers. Any product that may be evaluated in this article, or claim that may be made by its manufacturer, is not guaranteed or endorsed by the publisher.

## References

[1] Sahni M Bhandari V. Recent advances in understanding and management of bronchopulmonary dysplasia. F1000Res. (2020) 9:F1000. 10.12688/f1000research.25338.1 32704351 PMC7361502

[2] Jensen EA Schmidt B. Epidemiology of bronchopulmonary dysplasia. Birth Defects Res A Clin Mol Teratol. (2014) 100:145–57. 10.1002/bdra.23235 24639412 PMC8604158

[3] Philpot PA Bhandari V. Predicting the likelihood of bronchopulmonary dysplasia in premature neonates. Expert Rev Respir Med. (2019) 13:871–84. 10.1080/17476348.2019.1648215 31340666

[4] Gentle SJ Lal CV. Predicting BPD: lessons learned from the airway microbiome of preterm infants. Front Pediatr. (2020) 7:564. 10.3389/fped.2019.00564 32117822 PMC7011099

[5] Roth DE Abrams SA Aloia J Bergeron G Bourassa MW Brown KH. Global prevalence and disease burden of vitamin D deficiency: a roadmap for action in low- and middle-income countries. Ann N Y Acad Sci. (2018) 1430:44–79. 10.1111/nyas.13968 30225965 PMC7309365

[6] Bärebring L Bullarbo M Glantz A Hulthén L Ellis J Jagner Å. Trajectory of vitamin D status during pregnancy in relation to neonatal birth size and fetal survival: a prospective cohort study. BMC Pregnancy Childbirth. (2018) 18:51. 10.1186/s12884-018-1683-7 29439677 PMC5812027

[7] Yu L Guo Y Ke HJ He YS Che D Wu JL. Vitamin D status in pregnant women in southern china and risk of preterm birth: a large-scale retrospective cohort study. Med Sci Monit. (2019) 25:7755–62. 10.12659/MSM.919307 31617502 PMC6816329

[8] Kazzi SNJ Karnati S Puthuraya S Thomas R. Vitamin D deficiency and respiratory morbidity among African American very low birth weight infants. Early Hum Dev. (2018) 119:19–24. 10.1016/j.earlhumdev.2018.02.013 29518647

[9] Papalia H Samonini A Buffat C Gras E. des Robert C, Landrier JF, et al. Low vitamin D levels at birth and early respiratory outcome in infants with gestational age less than 29 weeks. Front Pediatr. (2022) 9:790839. 10.3389/fped.2021.790839 35127591 PMC8814585

[10] Lu T Liang B Jia Y Cai J Wang D Liu M. Relationship between bronchopulmonary dysplasia, long-term lung function, and vitamin D level at birth in preterm infants. Transl Pediatr. (2021) 10:3075–81. 10.21037/tp-21-494 34976773 PMC8649600

[11] Matejek T Navratilova M Zaloudkova L Malakova J Maly J Skalova S. Vitamin D status of very low birth weight infants at birth and the effects of generally recommended supplementation on their vitamin D levels at discharge. J Matern Fetal Neonatal Med. (2020) 33:3784–90. 10.1080/14767058.2019.1586873 30810408

[12] Onwuneme C Martin F McCarthy R Carroll A Segurado R Murphy J. The association of vitamin D status with acute respiratory morbidity in preterm infants. J Pediatr. (2015) 166:1175–80.e1. 10.1016/j.jpeds.2015.01.055 25919726

[13] Kim I Kim SS Song JI Yoon SH Park GY Lee YW. Association between vitamin D level at birth and respiratory morbidities in very-low-birth-weight infants. Korean J Pediatr. (2019) 62:166–72. 10.3345/kjp.2018.06632 30360037 PMC6528057

[14] Mao X Qiu J Zhao L Xu J Yin J Yang Y. Vitamin D and IL-10 deficiency in preterm neonates with bronchopulmonary dysplasia. Front Pediatr. (2018) 6:246. 10.3389/fped.2018.00246 30246004 PMC6137192

[15] Park HW Lim G Park YM Chang M Son JS Lee R. Association between vitamin D level and bronchopulmonary dysplasia: a systematic review and meta-analysis. PLoS ONE. (2020) 15:e0235332. 10.1371/journal.pone.0235332 32628705 PMC7337306

[16] Ge H Liu W Li H Zhang M Zhang M Liu C. The association of vitamin D and vitamin E levels at birth with bronchopulmonary dysplasia in preterm infants. Pediatr Pulmonol. (2021) 56:2108–13. 10.1002/ppul.25414 33878218

[17] Lykkedegn S Sorensen GL Beck-Nielsen SS Christesen HT. The impact of vitamin D on fetal and neonatal lung maturation. A systematic review. Am J Physiol Lung Cell Mol Physiol. (2015) 308:L587–602. 10.1152/ajplung.00117.2014 25595644

[18] Gil Á Plaza-Diaz J Mesa MD. Vitamin D: classic and novel actions. Ann Nutr Metab. (2018) 72:87–95. 10.1159/000486536 29346788

[19] Zhao Y Yu Y Li H Chang Z Li Y Duan Y. Vitamin D status and the prevalence of deficiency in lactating women from eight provinces and municipalities in China. PLoS ONE. (2017) 12:e0174378. 10.1371/journal.pone.0174378 28334009 PMC5363952

[20] Ge H Qiao Y Ge J Li J Hu K Chen X. Effects of early vitamin D supplementation on the prevention of bronchopulmonary dysplasia in preterm infants. Pediatr Pulmonol. (2022) 57:1015–21. 10.1002/ppul.25813 34989171

[21] Abrams SA. Vitamin D in preterm and full-term infants. Ann Nutr Metab. (2020) 76:6–14. 10.1159/000508421 33232955

[22] Bacchetta J Edouard T Laverny G Bernardor J Bertholet-Thomas A Castanet M. Vitamin D and calcium intakes in general pediatric populations: a French expert consensus paper. Arch Pediatr. (2022) 29:312–25. 10.1016/j.arcped.2022.02.008 35305879

[23] Saggese G Vierucci F Prodam F Cardinale F Cetin I Chiappini E. Vitamin D in pediatric age: consensus of the Italian Pediatric Society and the Italian Society of Preventive and Social Pediatrics, jointly with the Italian Federation of Pediatricians. Ital J Pediatr. (2018) 44:51. 10.1186/s13052-018-0488-7 29739471 PMC5941617

[24] Munns CF Shaw N Kiely M Specker BL Thacher TD Ozono K. et L. Global Consensus recommendations on prevention and management of nutritional rickets. J Clin Endocrinol Metab. (2016) 101:394–415. 10.1210/jc.2015-2175 26745253 PMC4880117

[25] Arundel P Ahmed SF Allgrove J Bishop NJ Burren CP Jacobs B. British Paediatric and Adolescent Bone Group's position statement on vitamin D deficiency. BMJ. (2012) 345:e8182. 10.1136/bmj.e8182 23208261

[26] Higgins RD Jobe AH Koso-Thomas M Bancalari E Viscardi RM Hartert TV. Bronchopulmonary dysplasia: executive summary of a workshop. J Pediatr. (2018) 197:300–8. 10.1016/j.jpeds.2018.01.043 29551318 PMC5970962

[27] Chen D Liu X Li J. Mechanical ventilation in neonatal respiratory distress syndrome at high altitude: a retrospective study from Tibet. Front Pediatr. (2019) 7:476. 10.3389/fped.2019.00476 31803698 PMC6877749

[28] Duke T. Neonatal pneumonia in developing countries. Arch Dis Child Fetal Neonatal Ed. (2005) 90:F211–9. 10.1136/adc.2003.048108 15846010 PMC1721897

[29] Nissen MD. Congenital and neonatal pneumonia. Paediatr Respir Rev. (2007) 8:195–203. 10.1016/j.prrv.2007.07.001 17868917

[30] Editorial Board Chinese Journal of Pediatrics the Subspecialty Group of Neonatology the Society of Pediatrics Chinese Medical Association. The neonatal mechanical ventilation routine. Zhonghua Er Ke Za Zhi. (2015) 53:327–30. 10.3760/cma.j.issn.0578-1310.2015.05.003 26080660

[31] Sweet DG Carnielli V Greisen G Hallman M Ozek E Plavka R. European consensus guidelines on the management of neonatal respiratory distress syndrome in preterm infants—2013 update. Neonatology. (2013) 103:353–68. 10.1159/000349928 23736015

[32] Working Working Group of Pediatrics Chinese Society of Parenteral and Enteral Nutrition Working Working Group of Neonatology Chinese Society of Pediatrics Working Working Group of Neonatal Surgery Chinese Society of Pediatric Surgery. CSPEN guidelines for nutrition support in neonates. Asia Pac J Clin Nutr. (2013) 22:655–63. 10.6133/apjcn.2013.22.4.21 24231027

[33] Sotunde OF Laliberte A Weiler HA. Maternal risk factors and newborn infant vitamin D status: a scoping literature review. Nutr Res. (2019) 63:1–20. 10.1016/j.nutres.2018.11.011 30824393

[34] Kassai MS Cafeo FR Affonso-Kaufman FA Suano-Souza FI Sarni ROS. Vitamin D plasma concentrations in pregnant women and their preterm newborns. BMC Pregnancy Childbirth. (2018) 18:412. 10.1186/s12884-018-2045-1 30348112 PMC6198501

[35] Thébaud B Goss KN Laughon M Whitsett JA Abman SH Steinhorn RH. Bronchopulmonary dysplasia. Nat Rev Dis Primers. (2019) 5:78. 10.1038/s41572-019-0127-7 31727986 PMC6986462

[36] Taylor SK Sakurai R Sakurai T Rehan VK. Inhaled Vitamin D: a novel strategy to enhance neonatal lung maturation. Lung. (2016) 194:931–43. 10.1007/s00408-016-9939-3 27614961 PMC5191914

[37] Wang P Tan ZX Fu L Fan YJ Luo B Zhang ZH. Gestational vitamin D deficiency impairs fetal lung development through suppressing type II pneumocyte differentiation. Reprod Toxicol. (2020) 94:40–7. 10.1016/j.reprotox.2020.03.008 32330513

[38] Sakurai R Singh H Wang Y Harb A Gornes C Liu J. Effect of perinatal vitamin D deficiency on lung mesenchymal stem cell differentiation and injury repair potential. Am J Respir Cell Mol Biol. (2021) 65:521–31. 10.1165/rcmb.2020-0183OC 34126864 PMC8641851

[39] Ahmad S Arora S Khan S Mohsin M Mohan A Manda K. Vitamin D and its therapeutic relevance in pulmonary diseases. J Nutr Biochem. (2021) 90:108571. 10.1016/j.jnutbio.2020.108571 33388351

[40] Mensink-Bout SM van Meel ER de Jongste JC Voortman T Reiss IK De Jong NW. Maternal and neonatal 25-hydroxyvitamin D concentrations and school-age lung function, asthma and allergy. The generation R study. Clin Exp Allergy. (2019) 49:900–10. 10.1111/cea.13384 30866115 PMC6850458

[41] Zhang X Luo K He X Chen P. Association of vitamin D status at birth with pulmonary disease morbidity in very preterm infants. Pediatr Pulmonol. (2021) 56:1215–20. 10.1002/ppul.25233 33331677

[42] Ramos-Navarro C Maderuelo-Rodríguez E Concheiro-Guisán A Pérez-Tarazona S Rueda-Esteban S Sánchez-Torres A. Risk factors and bronchopulmonary dysplasia severity: data from the Spanish Bronchopulmonary Dysplasia Research Network. Eur J Pediatr. (2022) 181:789–99. 10.21203/rs.3.rs-532632/v1 34596741

[43] Zhang J Luo C Lei M Shi Z Cheng X Wang L. Development and validation of a nomogram for predicting bronchopulmonary dysplasia in very-low-birth-weight infants. Front Pediatr. (2021) 9:648828. 10.3389/fped.2021.648828 33816409 PMC8017311

[44] Al Saleh Y Beshyah SA Hussein W Almadani A Hassoun A Al Mamari A. Diagnosis and management of vitamin D deficiency in the Gulf Cooperative Council (GCC) countries: an expert consensus summary statement from the GCC vitamin D advisory board. Arch Osteoporos. (2020) 15:35. 10.1007/s11657-020-0709-8 32124080

[45] Fukumoto S Ozono K Michigami T Minagawa M Okazaki R Sugimoto T. Pathogenesis and diagnostic criteria for rickets and osteomalacia-proposal by an expert panel supported by the Ministry of Health, Labour and Welfare, Japan, the Japanese Society for Bone and Mineral Research, and the Japan Endocrine Society. J Bone Miner Metab. (2015) 33:467–73. 10.1007/s00774-015-0698-7 26197863

[46] Golden NH Abrams SA. Committee on Nutrition. Optimizing bone health in children and adolescents. Pediatrics. (2014) 134:e1229–43. 10.1542/peds.2014-2173 25266429

[47] Abrams SA. Committee on Nutrition. Calcium and vitamin d requirements of enterally fed preterm infants. Pediatrics. (2013) 131:e1676–83. 10.1542/peds.2013-0420 23629620

[48] Bronsky J Campoy C Braegger C. ESPGHAN/ESPEN/ESPR/CSPEN working group on pediatric parenteral nutrition. ESPGHAN/ESPEN/ESPR/CSPEN guidelines on pediatric parenteral nutrition: vitamins. Clin Nutr. (2018) 37:2366–78. 10.1016/j.clnu.2018.06.951 30100105

[49] Fort P Salas AA Nicola T Craig CM Carlo WA Ambalavanan N. A comparison of 3 vitamin D dosing regimens in extremely preterm infants: a randomized controlled trial. J Pediatr. (2016) 174:132–8.e1. 10.1016/j.jpeds.2016.03.028 27079965 PMC4925243

[50] Byun SY Bae MH Lee NR Han YM Park KH. Association between vitamin D deficiency at one month of age and bronchopulmonary dysplasia. Medicine. (2021) 100:e27966. 10.1097/MD.0000000000027966 35049200 PMC9191292

